# Ocean acidification affects microbial community and invertebrate settlement on biofilms

**DOI:** 10.1038/s41598-020-60023-4

**Published:** 2020-02-24

**Authors:** Katie S. Nelson, Federico Baltar, Miles D. Lamare, Sergio E. Morales

**Affiliations:** 1 Department of Marine Science, University of Otago, PO Box 56, Dunedin, 9054 New Zealand; 2 NIWA/University of Otago Research Centre for Oceanography, PO Box 56, Dunedin, 9054 New Zealand; 3 Department of Microbiology and Immunology, University of Otago, PO Box 56, Dunedin, 9054 New Zealand; 4 Department of Functional and Evolutionary Ecology, University of Vienna, A-1090 Vienna, Austria

**Keywords:** Microbial ecology, Climate-change impacts, Marine biology

## Abstract

Increased atmospheric CO_2_ is driving ocean acidification (OA), and potential changes in marine ecosystems. Research shows that both planktonic and benthic communities are affected, but how these changes are linked remains unresolved. Here we show experimentally that decreasing seawater pH (from pH 8.1 to 7.8 and 7.4) leads to reduced biofilm formation and lower primary producer biomass within biofilms. These changes occurred concurrently with a re-arrangement of the biofilm microbial communities. Changes suggest a potential shift from autotrophic to heterotrophic dominated biofilms in response to reduced pH. In a complimentary experiment, biofilms reared under reduced pH resulted in altered larval settlement for a model species (*Galeolaria hystrix*). These findings show that there is a potential cascade of impacts arising from OA effects on biofilms that may drive important community shifts through altered settlement patterns of benthic species.

## Introduction

Since the start of the industrial revolution, an additional 555 Pg of carbon has been released into the atmosphere, of which 155 Pg C (≈30%) has entered the ocean. This process is predicted to decrease seawater pH by 0.2 to 0.4 units on average by the year 2100^1^. It is estimated that average surface ocean pH has already decreased from near 8.25 to 8.1 over the past 250 years and is forecasted to decrease to near or below 7.85 by the end of the century^1^. Acidification of the oceans is expected, and has been shown experimentally, to affect marine ecosystems in a myriad of ways with stimulative, inhibitive, or neutral effects depending on organism or location^2–5^.

Complex lifecycles in most marine invertebrates involve a long-lived benthic adult stage, and a shorter-lived planktonic larval stage that are thought to be most susceptible to climate change^6–8^. At the completion of the free-living larval stage, the competent larvae of benthic species must attach to a substrate (settle) and metamorphose. The transition is likely to affect settlement success of calcifying species under ocean acidification regimes; the first is a direct effect where lowered pH and lower saturation states of calcium carbonate (calcite and aragonite) ions which reduce calcification during the transition from planktonic to benthic life stages^9,10^; the second is an indirect effect through the disturbance of settlement cues between biofilms and larvae^11^. While evidence supports impacts of OA on calcification, recent work shows that these effects are likely driven by changes in saturation state and not directly by pH^12,13^. Less is known on the responses across life-history stages to OA scenarios that might be related to a loss of interaction (settlement cues) between larvae and microbial communities. While reduced pH has been linked to decreases in the settlement success of vermetids^14^, corals^14–18^, sea stars^11^, and sea urchins^19^, our understanding regarding the OA effects on the settlement processes of marine invertebrates remains limited. In this respect, a recent review by Espinel *et al*.^20^ found less than 50 published studies, with the majority indicated neutral or negative changes in settlement under reduced pH. Within these studies, only a small number examined the outcomes of pH induced changes of substrates (biofilms and CCAs) on settlement, and these indicated a reduction in settlement rate in taxa such as coral and sea stars^20^.

Settlement success has in part been attributed to biofilm recognition or quorum sensing^21,22^. These cues have been strongly linked to biofilm bacteria of the genus *Pseudoalteromonas*^22,23^ and the production of brominated compounds, with their effect potentially modified by factors including carotenoids^24^. It is likely, however, that a large number of microorganisms and metabolites are involved in promoting settlement of marine species, yet to be described. Altered settlement rates due to OA driven changes in biofilm communities and will be important as they could shape the future distributions, abundances and ecology of marine benthic communities. In addition, changes in settlement may impact the sustainability of species that are cultured or harvested, where recruitment and the supply of juveniles for recruitment and on-growing may be reduced.

The present research seeks to better understand the effects of reduced pH on the development of microbial biofilm communities, and the potential effects of changes in biofilms on the settlement of marine larvae. This is examined in two independent, but complimentary experiments. Firstly, we developed biofilms in flow-through aquaria for up to 69 days at ambient (pH 8.1) or reduced seawater pH (7.8 and 7.4) to determine their general characteristics (biomass, chlorophyll and carotenoid levels) and associated microbial community (diversity, composition). Secondly, we later assayed biofilms for settlement cues using competent larvae of a common polychaete tubeworm (*Galeolaria hystrix*). This species is an abundant, intertidal suspension feeder that typically settle as individuals in the shallow waters of low tidal zones at Portobello Beach, New Zealand^25^. The complex, heavily biofilmed and highly variable environment in which *G*. *hystrix* thrives made this serpulid polychaete a preferable model species to illustrate the cascading effects of ocean acidification on micro- to macro-community formation.

## Results

### Environmental seawater

**Figure 1:**
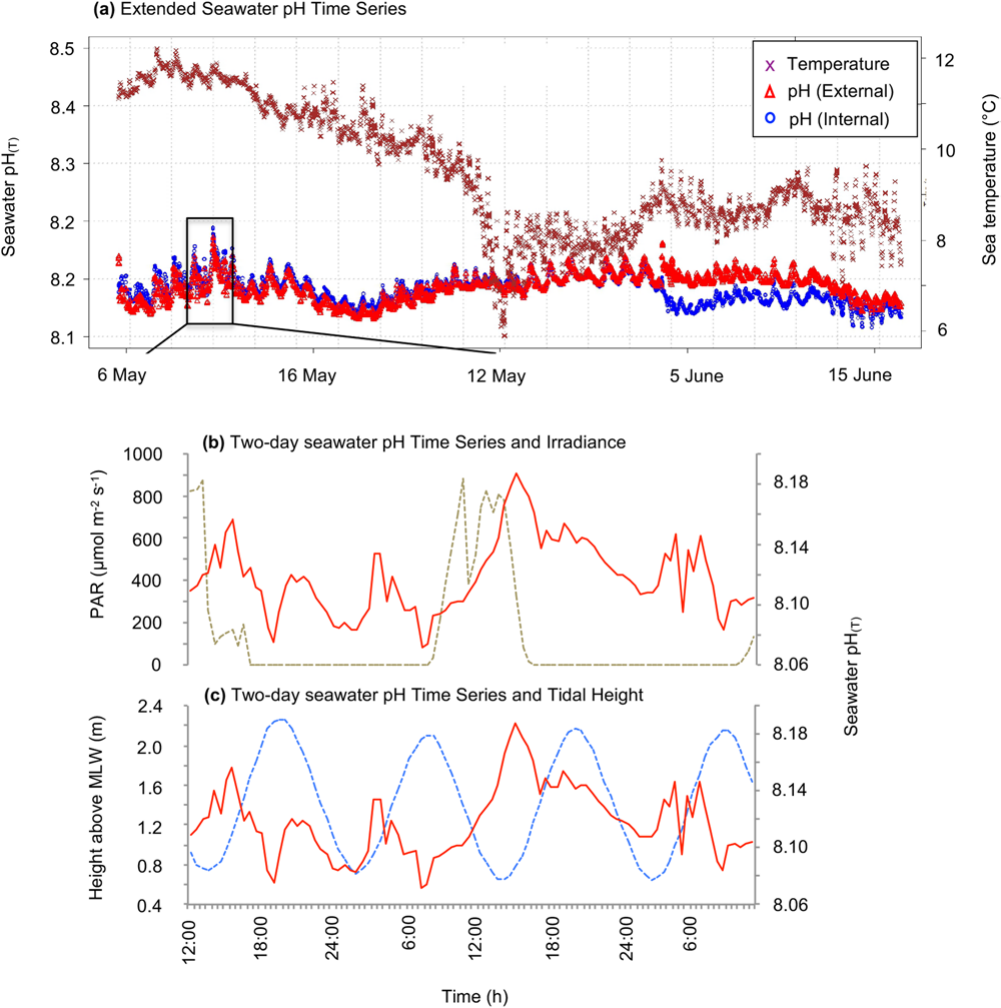
(**a**) Seawater pH_(T)_ and sea temperature (°C) from 42-day deployment of the SeaFET at Portobello Marine Lab (PML) Wharf, Otago Harbour at 1 m depth. (**b**) Seawater pH and PAR (photosynthetically active radiation, 700–400 nm) light time series at PML from 9 to 11 May 2015. (**c**) Seawater pH and tidal height time series at PML from 9 to 11 May 2015. The pH (Internal) and pH (External) are the internal and external reference electrode measurements, respectively, which are equivalent when the instrument is correctly calibrated for accurate measurement.

Over the 42-day deployment of the SeaFET in Otago Harbour, the seawater pH ranged from 8.04 to 8.19  pH units (Fig. 1a). Within this period a strong diel pattern was detected, with single fluctuations ranging up to 0.12  pH units (max = 8.19, min = 8.07). The daily variation in pH is most tightly coupled with daily light cycles (Fig. 1b), with seawater pH increasing from an overnight minimum and reaching a peak mid-afternoon, 2–3  hours after maximum mid-day irradiance. In contrast, tidal water exchange (which is semidiurnal at our study site) has a secondary and smaller influence on pH (Fig. 1c), with an incoming tide and tidal mixing associated with periods of variable and increasing pH.

### Experiment 1: Biofilm development under pH treatment

**Figure 2:**
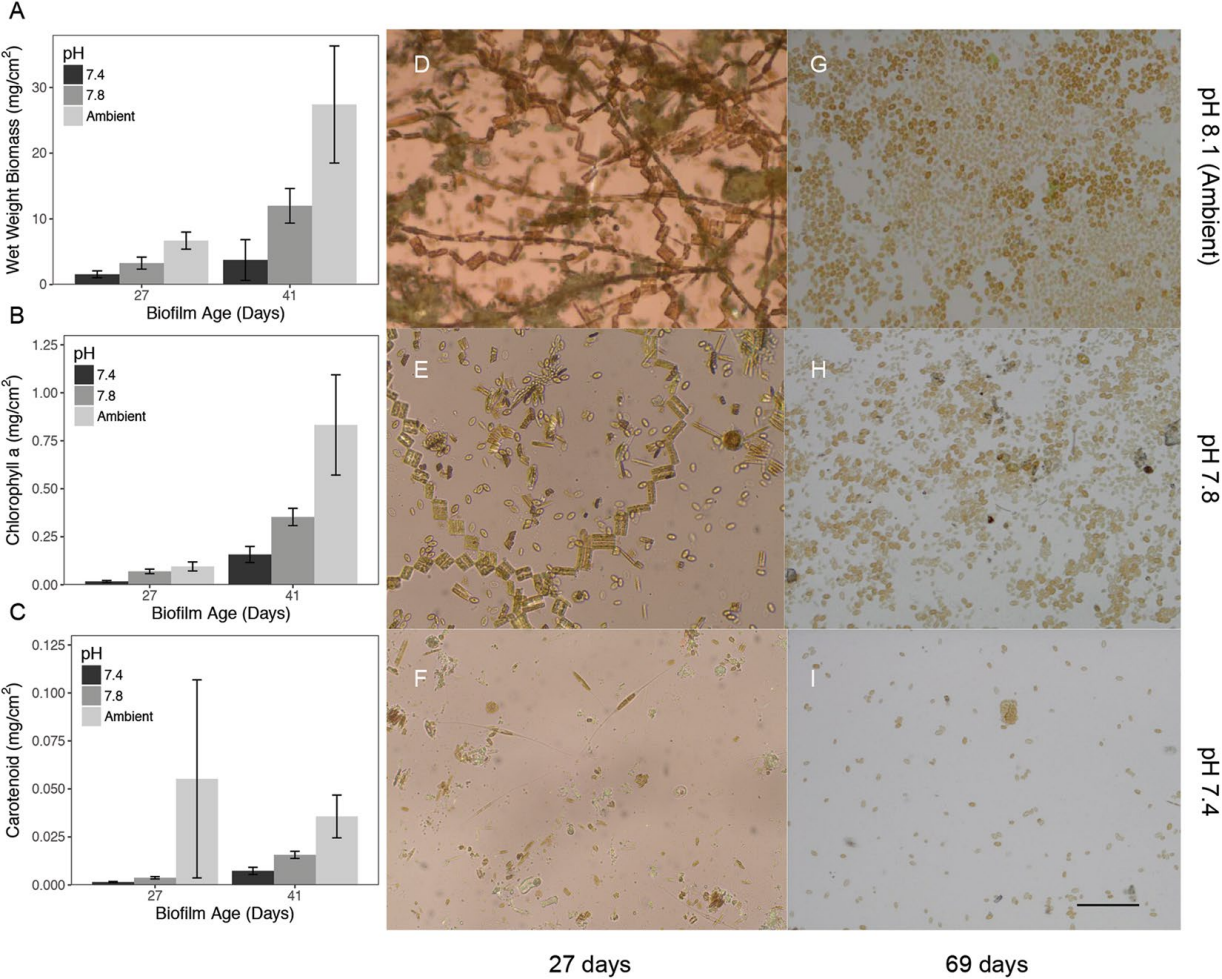
Effects of ocean acidification on biofilm development. (**A**–**C**) Changes in biomass and pigments associated to primary producers in response to pH treatment for young and old biofilms (n = 5 for each bar). (**D**–**I**) Representative images for biofilms at different pH for young and old biofilms. Ambient pH_NIST_ was 8.1. Scale bar 50 μm.

Biofilm wet weight biomass was significantly (ANOVA, F_2,9_ = 5.419 p < 0.05) different among pH treatments, with a more pronounced difference in older biofilms (Fig. 2). Biomass increased 12-fold under ambient pH conditions from young to older biofilms, while the increase over time was less pronounced under both reduced pH treatments. The biomass of old biofilms measured in the slides was 7-fold lower when these were developed at the lowest pH than at ambient conditions. This trend was consistent with the (5-fold) lower chlorophyll-a concentration for both young (ANOVA, F_2,9_ = 6.592, p < 0.05) and older biofilms (ANOVA, F_2,9_ = 12.725, p < 0.05), under the pH 7.4 treatment (Fig. 2). A similar trend was observed for carotenoid concentrations (Fig. 2) in young (ANOVA, F_2,9_ = 5.949, p < 0.05) and old biofilms (ANOVA, F_2,9_ = 14.001, p < 0.05).

### Microbial community analysis

**Figure 3:**
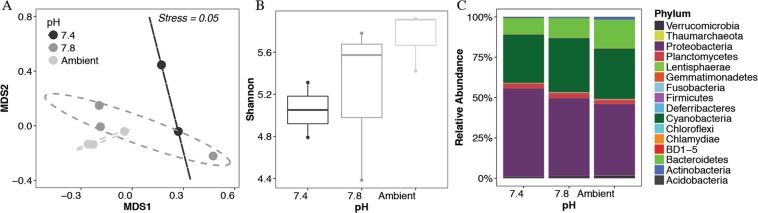
Microbial community response of 27-day old biofilms to ocean acidification. (**A**) Differences in communities, (**B**) Shannon diversity and (**C**) composition. Ambient pH was 8.1. Supplemental information and full data can be found in Figs. S1–4.

**Figure 4:**
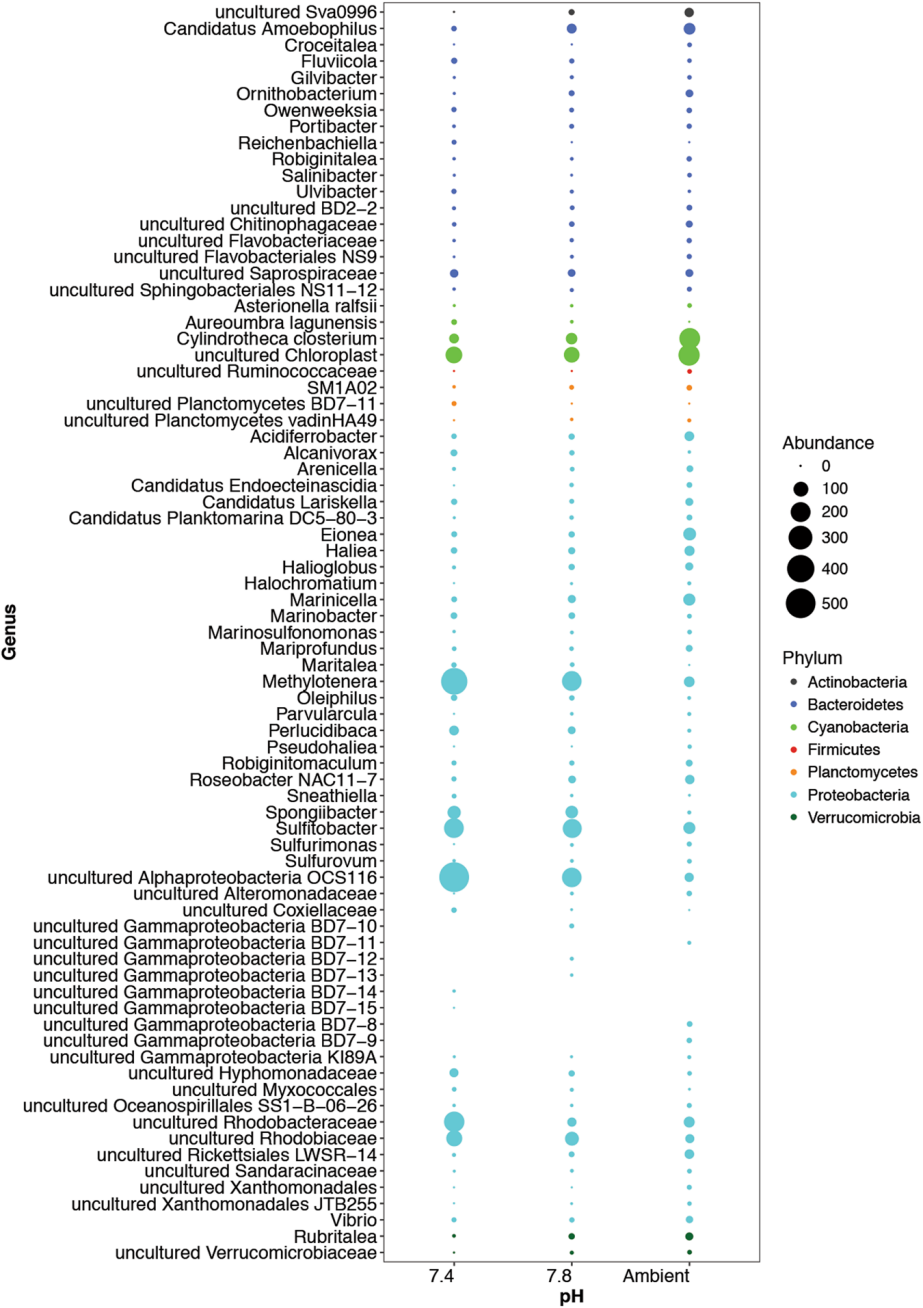
Summary of significantly affected microbial genera based on OTUs correlated to pH. Data represents 198 OTUs with a significant (p < 0.05) Spearmans correlation (Rho <−0.5 and >0.5).

Reduced pH also led to clear and simultaneous shifts in microbial communities (Fig. 3) for both young and old biofilms (Figs. 3 and 4, Supplementary Table 3). In young biofilms (<30 days old), microbial communities observed under both pH scenarios were statistically distinct from those raised in ambient pH (Fig. 3A and Supplementary Fig. 4) (ANOSIM: 16S, R = 0.54, p = 0.04; invertebrates, R = 0.49, p < 0.01), with the lowest observed variance in community changes at the lowest pH. Despite changes in community structure and a trend of reduced alpha diversity at lower pH for microbial communities (Fig. 3B), no significant differences (ANOVA p > 0.05) in diversity (both richness and Shannon diversity) were observed among microbial communities. A re-arrangement was also observed at a broad taxonomic level in the microbial community with fold decreases in Actinobacteria (−2.8x), Bacteroidetes (−1.7x), Firmicutes (−3.3x), Fusobacteria (−1.5x), Gemmatimonadetes (−2.2x), Nitrospiraea (−8x), Proteobacteria (−1.3x) anderrucomicrobia (−2x). In contrast, fold increases were observed for BD1–5 (3.5x) and Planctomycetes (1.4x). Not all microbial phyla responded in gradual fashion, however, with pH 7.8 being preferred (e.g. Candidate division BRC1) or avoided (e.g. Chlamydiae) by certain taxa. These groups were detectable across various pH levels, although certain groups went from undetected to regularly detected under OA conditions (Tenericutes, Candidate division SR1, NPL-UPA2, Tenericutes), and others were no longer detected at reduced pH (Deinococcus-Thermus).

While patterns at high taxonomic levels (phylum) suggest strong effects for certain microbial groups, we aimed to identify specific organisms (operational taxonomic units; OTUs) significantly associated with changing pH. A Spearmans correlation against pH identified 198 OTUs with strong (p < 0.05, Rho < −0.5 and >0.5) responses to pH (Fig. 4, Supplementary Figs. 5–7). Within responsive OTUs, certain taxa were negatively influenced by pH (Acidobacteria, Actinobacteria, Bacteroidetes, Cyanobacteria, Firmicutes, Gemmatimonadetes, Planctomycetes, and Verrucomicrobia) while others were positively affected (Chlamydiae, Lentisphaerae, and Proteobacteria). Interestingly the number of responsive organisms was highly variable across phyla with most organisms belonging to the Proteobacteria. Within this phyla, both positively (e.g. *Methylotenera*) and negatively (e.g. *Haliea*) affected organisms were detected. While OTUs closely associated to the gammaproteobacterial, *Pseudoalteromonas* were not detected, organisms within the gammaproteobacteria were amongst the most commonly associated with changes in pH, with 21 representatives from the Order Alteromonadales (were *Pseudoalteromonas* belongs), including 17 organisms negatively responsive to a decrease in pH. These low pH sensitive organisms (Spearmans correlation to pH > 0.72) included members of the genera *Haliea*, *Eionea*, *Halioglobus*, Candidatus Endobugula, *Pseudohaliea*, OM60(NOR5) clade, and several uncultured groups. Overall, this represents a gradual shift in the biofilm from a primary producer dominated community (e.g. Cyanobacteria and diatoms [Cylindrotheca]) to a heterotrophic (e.g. *Methylotenera* and Roseobacter OCS116 clade) dominated one, and is consistent with the observed decrease in biomass and pigments of primary producers (see Fig. 2).

### Experiment 2: Settlement assays on biofilms developed under pH treatment

**Figure 5:**
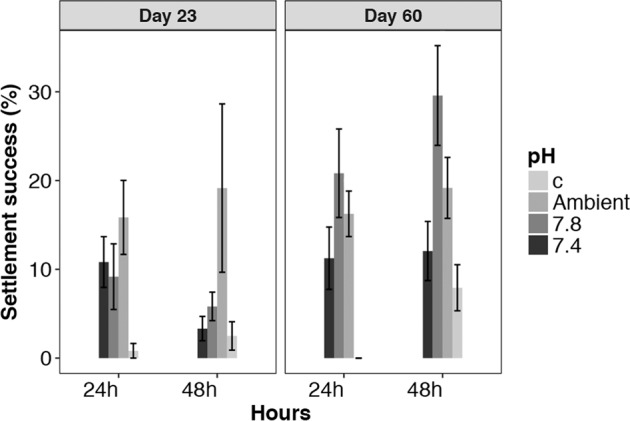
Settlement success of the model invertebrate (tubeworm) *Galeolaria hystrix* on biofilms grown at different pH. (**a**) The proportion of competent larvae settled 24 h and 48  h after being offered 23-day old biofilms developed under pH_NIST_ 7.4, 7.8 and 8.1. (**b**) The proportion of competent larvae settled 24 h and 48  h after being offered 60-day old biofilms developed under pH_NIST_ 7.4, 7.8 and 8.1. For each settlement experiment a control (sterile glass slide) was used to determine non-selective settlement.

Average *Galeolaria hystrix* settlement success ranged from 0.8% on clean control slides, up to 29% observed on 60-day old biofilms raised in pH 7.8 (Fig. 5). On 23-day old biofilm, a two-way ANOVA indicated a significant difference in settlement rate among pH treatments (F_(3, 31)_ = 8.838, p < 0.001). A Tukey’s Post hoc test indicated settlement was significantly greater on the ambient and pH 7.8 biofilms than on the control slides, but the difference was not significant among the pH treated biofilms, or between pH 7.4 and the control slides. This difference was consistent at both 24 and 48 h during the settlement assay.

On 60-day old biofilm (Fig. 5), a two-way ANOVA indicated settlement was significantly greater (F_(1, 63)_ = 7.479, p = 0.008) at 48  h than at 24  h, and was significantly different (F_(3, 63)_ = 19.614, p < 0.001) among pH treatments/control. A Tukey’s Post hoc test indicated settlement was significantly lower on the control slides. Within the pH treatments, settlement was not significantly different between the ambient and pH 7.8, nor between the ambient and pH 7.4. Settlement was, however, significantly greater on pH 7.8 compared with pH 7.4, with this pattern of settlement among pH treatments consistent at both 24 h and 48 h (Time × pH, F_(3, 63)_ = 1.624, p = 0.194).

## Discussion and Conclusions

Ocean acidification (OA) will alter marine biological processes, and while a wide range of individual responses are reported^4^, we aimed to address currently unresolved questions related to impacts across interactive trophic or taxonomic levels. In this study, using two independent but complimentary experiments, we showed that even a short term reduction in seawater pH (<69 days) can influence biofilm development and microbial community composition, and that modified biofilms may alter settlement rates in invertebrate larvae such as *Galeolaria hystrix*.

### Environmental pH variability and experimental treatments

In order to conduct ecologically meaningful *in vitro* ocean acidification experiments, relevant pH targets must consider site/ecosystem-specific carbonate systems and natural pH fluctuations of *in situ* habitats. Our *in situ* measurements show that seawater pH at our study, and where our biological experiments were conducted, has a relatively high degree of variability, over relatively short time scales (i.e. hours). This variability is associated with diurnal light cycles driving the photosynthetic uptake of CO_2_ (and a resulting reduction in pH) during daylight, while semi-diurnal tidal cycles mix water at the site with relatively high pH coastal seawater. Our observations of short term (hourly) and longer-term variability are consistent with measurements of pH for nearshore and estuarine environments^26^ where pH varies on the order of 0.2 to 0.3  pH units associated with biological activity and water exchange.

Over the measurement period, the average seawater pH_T_ (pH total scale) at the *in situ* collection site at Portobello Marine Laboratory was pH_T_ 8.13 ± 0.01 and was consistent with the 2010 global open-ocean estimate of pH_T_ 8.07 (NOAA, 2010). Therefore, the near-future, 2100 IPCC^1^ projection of pH_NIST_ 7.8^27^ was an appropriate near-future pH target. Although current research supports a pH of approximately pH < 7.5 if atmospheric CO_2_ reaches levels of 1900 ppm by 2300^1,27^, observed diel fluctuations (up to pH_T_ 0.116, Fig. 1) justified a slightly reduced target of pH_NIST_ 7.4 (pH National Institute of Standards and Technology scale) as a more ecologically relevant, extreme 2300  pH target. These pH targets are consistent with a number of *in vitro* studies^16,18,28–31^ and overlap with a number of *in situ* studies at CO_2_ vent sites^32–37^ allowing for comparison among observations.

### Experiment 1: Microbial community responses to reduced pH

In the first experiment, we observed shifts in biofilm community and structure across reduced pH suggesting a loss of cells, however it does not indicate a loss of diversity or potential functions encoded within biofilm organisms. Instead we saw a re-arrangement of the microbial community already detectable at a broad taxonomic level. The observed negative effect of OA of *Nitrospiraea* and *Actinobacteria* in this study is consistent with the decrease of *Nitrospiraea* sequences observed in response to natural *p*CO_2_ increases in coastal sediments along natural CO_2_ gradients at a volcanic vent in Papua New Guinea^38^. However, contrasting results were reported for Bacteriodetes and Proteobacteria in response to pH; in our study we found that they decreased in response to OA whereas Raulf *et al*.^38^ found a linear increase in relative sequence abundance with decreasing pH along their pH gradient. Consistent to our results, a decrease of Bacteroidetes sequence in response to OA was also found in microbial biofilms of crustose coralline algae^16^. These results could indicate differences in the response to OA of microbial communities living on sediments versus on other solid surfaces, or a differences related to the experimental conditions (i.e., mesocoms experiment vs natural *p*CO_2_ gradient). Nevertheless, the confirmation of *Nitrospiraea* and *Actinobacteria* changes suggests a key role of these organisms in the light of the responses of biofilm microbial communities to OA.

It is important to acknowledge that while certain taxa decreased, others had positive responses to changes in pH, including several groups from within the proteobacteria (e.g. Methylotenera, Sulfitobacter and unclassified alphaproteobacteria from the OCS116 cluster). Further studies are needed to confirm which key members of microbial biofilm communities are likely to have consistent responses to climate change pressures such as OA, and this would include examining multiple stressors simultaneously (e.g. pH, temperature, etc.). For example, it has been shown that in planktonic microbial communities the combination of OA and temperature^39^, or OA and eutrophication^40^ can select for specific members of the microbial communities which might differ from OA alone.

### Experiment 2: Biofilm modification and larval settlement

In the second experiment, settlement of *Galeolaria* was significantly different on glass slides that had been developed under the three pH treatments. The most likely cause of this pattern is a change in settlement cues associated with biofilms that had modified physical or biological/chemical characteristics. A loss of microorganisms known to trigger settlement^21,23^, as well as the reduction in other factors (e.g. carotenoids) known to enhance settlement^24^ suggest that OA scenarios, especially conditions such as acidification to pH 7.4, could lead to regime shifts that can alter key ecosystems processes such as recruitment and their services^41^.

While it is clear that microbes contribute to chemotaxis and settlement success^21,23^, and that acidification can influence invertebrate settlement^15–18^, a definitive link between these two processes remains unresolved. Microbial biofilms are likely critical for settlement induction in marine species^42–44^, and changes in biofilm composition, such as those related to age^21,45,46^ or pH^16^, can affect settlement rates in marine invertebrates. For example, Webster *et al*.^16^ reported decreases in dominant microbial populations (Alphaproteobacteria and Bacteroidetes) and emergence of Proteobacteria as a result of lower seawater pH. This coincided with reduced coral larvae settlement, although an explicit mechanism (e.g. altered settlement on CCA is due to changes in the chemical inducer of the algae or altered associated microbes) was not identified.

## Conclusions

There is an important caveat when interpreting our findings, namely that biofilm microbial assessments and settlement experiments were carried out on separately reared biofilms. Nevertheless, our results are consistent with prior work and support the hypothesis that alterations in microbial biofilms are directly linked to both changes in settlement and abundance of invertebrate communities. This may represent a model scenario to OA, but further studies are needed to directly provide a mechanism to explain how settlement cues may be influenced. Observed shifts in the settlement of *Galeolaria hystrix* suggest that changes in community structure for invertebrates were at least in part linked to changes in microbial biofilm composition. This has broader implications as altered settlement and reduction in biofilms could drive important changes in benthic communities’ composition and distribution.

Presently, there is limited information on the potential effects of OA on the settlement process^20,47^. Mostly, it has shown that OA has a negative impact on settlement in marine invertebrates, however these conclusions are based on relatively few taxa, and mostly within the Cnidaria and Echinoderm phyla. In addition, settlement is a complex process that involves larval supply and behaviour, substrate characteristics and early post-settlement growth^8,48^, and the relative importance of OA-induced changes in each step required further research^20^. Similarly, settlement characteristics are likely species-species specific, with, for example in the degree of substrate selectivity. The present study provides investigations of the effects of OA on settlement substrates and settlement efficiency, and may provide the basis for future studies across a broader range of taxa.

## Methods

Detailed methods are provided in the supplementary materials including experimental set up, sea water chemistry, microbial community analyses, larval rearing and statistical analyses.

### Environmental pH measurements

*In situ* seawater pH_T_ was measured using a SeaFET ocean pH sensor (Sea Bird Scientific, USA) deployed continuously for 42 days from 4 May to 15 June 2015 at 1.0  m depth, coinciding with our period of Experiment 1. The SeaFET instrument utilizes both an internal and an external reference electrode for pH measurements (i.e. pH (Internal) and pH (external), respectively) and if the instrument is correctly calibrated and there will be close agreement between both pH measurement (SeaFET user manual, Sea Bird Scientific Document SeaFET170601, March 2019). The SeaFET was set to record an average pH every 30  minutes from 20 rapid recordings containing a burst of 30 measurements. Post-deployment processing is required to calculate seawater pH, and this was made using SeaFETCom software version 1.2. These calculations use salinity (recorded independently at an average of 35 PSU) and the ambient sea temperature (°C) simultaneously recorded by the SeaFET. Calculated pH over time was graphed using R-code developed by Andrew Marriner, Ocean Atmosphere Technician at National Institute of Water and Atmospheric Research.

### Experiment 1: Biofilm development under pH treatment

Biofilms were developed in flow-through seawater aquarium supplied with the desired pH seawater. Each pH level had an independent 70  L header tank in which the supplied seawater pH was adjusted, before flowing into four replicate 5 L aquaria which contained the biofilm slides (see Supplementary Fig. S8). The three pH treatments were ambient pH_NIST_ 8.1 (*p*CO_2_ = 384 ppm), pH_NIST_ 7.8 (near future, 2100, *p*CO_2_ = 1108 ppm) and pH_NIST_ 7.4 (extreme, 2300, *p*CO_2_ = 2465 ppm). Biofilms were developed on glass microscope slides suspended below the water’s surface at a 45° angle perpendicular to flow-through direction. Samples for microbial community analysis were collected from an initial set of incubated slides incubated for up to 69 days in Experiment 1, while a second development of slides up to 60-days period was used for development of biofilms for settlement assays in Experiment 2. Biofilm wet weight biomass and microbial community analysis were collected from Experiment 1, and chlorophyll-a and carotenoid analysis were collected from Experiment 2. Biofilm and pigment analysis were made for 5 replicate slides from each treatment.

### Experiment 1: Microbial biofilm characteristics and community analysis

Biofilm material was carefully removed from both sides of the lower 15  cm^2^ section of each slide (total area = 30  cm^2^) using a sterilised metal laboratory spatula. Samples were stored at −4 °C until transfer to the lab and stored until processing at −20 °C. Further processing was performed as previously described^49^. Total DNA was extracted from each individual biofilm sample using a MoBio PowerSoil DNA Isolation Kit (MO BIO Laboratories Inc., Solana Beach, CA, USA) following manufacturer’s protocol with the following modification: Bead-beating (2 × 15 s) cycles was performed using a 2010 GenoGrinder (SPEX SamplePrep, Metuchen, NJ, USA). After extraction, a Nanodrop Spectrophotometer (Thermo Fisher Scientific, Waltham, MA, USA) was used to assess DNA quantity and quality.

The 16S rRNA gene amplicon sequencing was performed using primers 515 F/806 R (V4 region of the 16S gene) and the Earth Microbiome Project protocol (Version 4_13)^50^. All samples were sequenced on a single Illumina MiSeq run. Sequences were first processed in Qiime (version 1.9.1) using default parameters^51^ including minimum read length of 75  bp, min number of consecutive high quality base calls to include a read as a fraction of the input read length of 0.75, Phred quality score of 3, no ambiguous bases allowed, and no mismatches allowed in primer sequence^52^. All sequences kept for analysis were 151  bp. Sequence clustering (97% sequence similarity) into Operational Taxonomic Units (OTUs) was done using the SILVA (version 119) reference library^53^ and UCLUST^54^ following the open-reference Operational Taxonomic Unit (OTU) picking protocol. Taxonomy assignments were done using BLAST against the SILVA database (max-e value = 0.001)^55^. Subsampling and rarefactions (10 times) were performed to equal read depths of 8,000 per sample, and samples below that threshold were removed. After rarefaction, all 10 OTU tables were merged and exported for further processing in R^56^. The rarified OTU table (in biom format) was processed using the phyloseq package^57^. To account for the multiple rarefication (10 total) abundances, a mean was calculated (dividing by 10) and results were rounded to whole integers using the transform sample_counts() command. Taxa (OTUs) with less than 1 count were deleted using the prune_taxa() command. Alpha diversity (Shannon and richness) were calculated using the estimate_richness() command.

The NMDS plot was created using a Bray-Curtis distance matrix through “phyloseq” and “vegan”^58^ packages. Significant treatment and age effects were determined using an Anosim test. To determine samples forming statistically significant groups, a cluster analysis was performed using the pvclust package (method = Ward; distance matrix = Bray-Curtis; bootstrap value, n = 1000)^59^. Significant groups (representing 95% confidence) were marked with boxes (red). All data analyzed in this paper along with analysis code can be found at: https://github.com/semorales/Nelson_OA_2019.

### Experiment 2: Biofilm modification and larval settlement

We examined the effect of biofilms developed under experimental pH conditions on the settlement success of the polychaete tubeworm *Galeolaria hystrix*. Settlement assays were conducted using *G*. *hystrix* larvae reared to competency using methods described by Nelson *et al*.^25^, and placed with 23-day old and 60-day old biofilms developed on glass slides in the flow-through system during experimental period 2. Settlement was measured and scored at 24 h and 48 h after the introduction of competent larvae to the substrates. Clean glass slides with no biofilm developed on the surface was used as a negative control for non-specific background settlement.

## Supplementary information

Supplementary Information.

Supplementary Methods.
