# Supplementary material for: Ocean acidification affects microbial community and invertebrate settlement on biofilms
Source: Sci Rep. 2020 Feb 24;10:3274. doi: 10.1038/s41598-020-60023-4 (PMC7039980; doi:10.1038/s41598-020-60023-4)
Supplement: Supplementary file 1 — Supplementary Information. [file 41598_2020_60023_MOESM1_ESM.docx]

**Ocean acidification affects microbial community and invertebrate settlement on biofilms**

Katie Nelson, Federico Baltar, Miles Lamare and Sergio E. Morales

**Supplementary Table 1.** *In situ* carbonate chemistry replication data to confirm collection method

**Supplementary Table 2.** Average (±SE) seawater chemistry measurements of biofilm development treatments

**Supplementary Table 3**. ANOSIM results for Figure 2

**Supplementary Figure 1.** Non-metric multidimensional scaling (NMDS) plot comparing microbial community changes across age and pH treatment.

**Supplementary Figure 2.** Stress plot for Figure S3 (microbial data by age and pH).

**Supplementary Figure 3.** Stress plot for micro data by pH day 27 only (Fig3b)

**Supplementary Figure 4.** Cluster dendrogram with AU/BP values (%) 90% confidence boxes suggest 2 clusters (Fig 3a) with pH 7.4 biofilms significantly different from all others, and ambient (pH 8.13) biofilms clustering separately from pH 7.8 ones.

**Supplementary Figure 5.** ANOSIM results for microbial community changes against pH.

**Supplementary Figure 6.** Summary (at Phylum and Class level) of significantly affected OTUs identified by Spearmans correlations (p < 0.5).

**Supplementary Figure 7.** Summary (Genus level) of the top 20 most abundant OTUs identified as significantly affected (by Spearmans correlations (p < 0.5)) by pH.

**Supplementary Figure 8.** Schematic illustration of tank flow through and settlement assay experimental design.

**Supplementary Figure 9.** Record of flow-through system treatment pH measurements

**Table S1.** Raw data of duplicate TA and DIC measurements and the effect on calculated carbonate parameters (pH_T_, *p*CO_2_, Ω_C_ and Ω_A_)*. Samples are labeled by location, duplicate number and date (2015).

|  | **TA**  ***μ* mol kg^-1^** | **DIC**  ***μ* mol kg^-1^** | **pH_T_** | ***p*CO_2_**  ***μ*atm** | **Ω_C_** | **Ω_A_** |
| --- | --- | --- | --- | --- | --- | --- |
| Intertidal 1 (23 Mar) | 2280.7 | 2084.7 | 8.04 | 428.91 | 3.40 | 2.18 |
| Duplicate 1 (23 Mar) | 2288.9 | 2083.2 | 8.04 | 408.24 | 3.55 | 2.27 |
| Subtidal 2 (4 May) | 2287.7 | 2087.7 | 8.06 | 375.03 | 3.42 | 2.18 |
| Duplicate 2 (4 May) | 2286.1 | 2088.3 | 8.06 | 379.37 | 3.39 | 2.16 |
| Intertidal 3 (4 Aug) | 2284.5 | 2067.8 | 8.16 | 287.44 | 3.66 | 2.31 |
| Duplicate 3 (4 Aug) | 2281.6 | 2068.3 | 8.16 | 292.35 | 3.60 | 2.28 |

* *In situ* measurements of pH on the total scale, pCO_2_ (partial pressure CO_2_), TA (total alkalinity), DIC (dissolved inorganic carbon) Ω_C_ (calcite saturation state) and Ω_A_(aragonite saturation state).

**Table S2.** Average (±SE) seawater chemistry measurements of biofilm development treatments for period 1 (P1) from May–June 2015 and period 2 (P2) from August–October 2015. Three water samples were taken over each time period, n=3. Temperature, salinity, TA and DIC were measured. pH_NIST_, pCO_2_, Ω_Ca_ and Ω_Ar_ were calculated for in situ temperatures (°C).

| **Treatment** (Target pH_NIST_) | **Temperature**  **(C°)** | **Salinity**  **(ppt)** | **pH_NIST_** | ***p*CO_2_**  *μ*atm | **TA**  *μ*mol kg^-1^ | **DIC**  *μ*mol kg^-1^ | | **Ω_Ca_** | | **Ω_Ar_** |
| --- | --- | --- | --- | --- | --- | --- | --- | --- | --- | --- |
| Ambient (P1) | 10.0  (1.3) | 33.4  (0.8) | 8.13 (0.01) | 413.30 (10.21) | 2214.2 (43.8) | 2052.1 (33.6) | 2.86 (0.18) | | 1.81 (0.12) | |
| Ambient (P2) | 10.2  (1.2) | 33.5  (0.2) | 8.20 (0.05) | 354.58 (43.51) | 2267.9 (10.5) | 2068.77 (23.3) | 3.42 (0.24) | | 2.17 (0.15) | |
| 7.8 pH (P1) | 10.0  (1.3) | 34.3  (0.7) | 7.81 (0.17) | 1036.38 (307.35) | 2214.5 (43.5) | 2184.5 (31.6) | 1.83 (0.84) | | 1.17 (0.54) | |
| 7.8 pH (P2) | 10.2  (1.2) | 33.9  (0.2) | 7.73 (0.05) | 1180.51 (171.28) | 2271.5 (9.6) | 2241.4 (19.8) | 1.29 (0.08) | | 0.82 (0.05) | |
| 7.4 pH (P1) | 10.0  (1.3) | 34.3  (0.7) | 7.43 (0.03) | 2317.26 (162.33) | 2239.6 (30.7) | 2299.2 (38.6) | 0.66 (0.03) | | 0.42 (0.02) | |
| 7.4 pH (P2) | 10.2  (1.2) | 33.6  (0.2) | 7.40 (0.04) | 2612.17 (324.26) | 2274.7 (8.7) | 2349.1 (18.9) | 0.62 (0.03) | | 0.39 (0.02) | |

* *In situ*measurements of pH on the NBS scale, pCO_2_ (partial pressure CO_2_), TA (total alkalinity), DIC (dissolved inorganic carbon) Ω_Ca_ (calcite saturation state) and Ω_Ar_ (aragonite saturation state.

Table S3. ANOSIM results for Figure 2.

|  | **Df** | **Sum Sq** | **Mean Sq** | **F value** | **Pr(>F)** |
| --- | --- | --- | --- | --- | --- |
| **pH** | 2 | 247182 | 123591 | 5.883 | **0.02** |
| **Biofilm_Age_days** | 1 | 82226 | 82226 | 3.914 | 0.08 |
| **pH:Biofilm_Age_days** | 2 | 8228 | 4114 | 0.196 | 0.83 |
| **Residuals** | 10 | 210094 | 21009 |  |  |

**Figure S1.** Non-metric multidimensional scaling (NMDS) plot comparing microbial community changes across age and pH treatment. (see Table S2 for ANOSIM results).

**Figure S2.** Stress plot for Figure S3 (microbial data by age and pH).

**Figure S3.** Stress plot for micro data by pH day 27 only (Fig3a).

**Figure S4.** Cluster dendrogram with AU/BP values (%) 90% confidence boxes suggest 2 clusters (Fig 3a) with pH 7.4 biofilms significantly different from all others, and ambient (pH 8.13) biofilms clustering separately from pH 7.8 ones.

**Figure S5.** ANOSIM results for microbial community changes against pH.

**Figure S6.** Summary (at Phylum and Class level) of significantly affected OTUs identified by Spearmans correlations (p < 0.5).

**Figure S7.** Summary (Genus level) of the top 20 most abundant OTUs identified as significantly affected (by Spearmans correlations (p < 0.5)) by pH. Plotted OTUs represent 86% of sequences associated with affected OTUs.

**Figure S8.** Schematic illustration of tank flow through and settlement assay experimental design.

**Figure S9.** Record of flow-through system treatment pHs at Portobello Marine Labortatory, Otago Harbour, New Zealand. Dates (2015) and the recorded pH_NIST_ readings for the pH_NIST_ 7.8 and pH_NIST_ 7.4 header tanks.
