# Supplementary material for: Ocean acidification affects microbial community and invertebrate settlement on biofilms
Source: Sci Rep. 2020 Feb 24;10:3274. doi: 10.1038/s41598-020-60023-4 (PMC7039980; doi:10.1038/s41598-020-60023-4)
Supplement: Supplementary file 2 — Supplementary Methods. [file 41598_2020_60023_MOESM2_ESM.docx]

**Methods**

*In situ pH measurement*

The physical and chemical seawater characteristics were recorded at two distinct locations off the Portobello Marine Laboratory (PML) Wharf, Otago, New Zealand (45° 49’ 40.7424” S, 170° 38’ 26.0160” E) at the collection site of the model species *Galeolaria hystrix.* *In situ* measurements were made between March to October 2015 from an intertidal site at approximately 0.1 m above the sea floor, and from an adjacent subtidal site at approximately 0.5 m below the surface. Discrete *in situ* seawater measurements of temperature (°C), salinity (ppt), dissolved oxygen (DO, mg/L) and pH_T_ were recorded periodically from March to October 2015 (interval range = every 3 to 21 days, mean = every 9 days). The frequency of measurements was limited by equipment availability and in some cases severe weather conditions limited access to the site. Two seawater samples were collected from each site; one in a 250 mL Duran Schott bottle (for dissolved inorganic carbon, DIC, analysis) and one in a 1 L PET bottle (for total alkalinity, TA, analysis). All samples were fixed with saturated mercuric chloride (HgCl_2_) for preservation and secured with parafilm for later laboratory analysis. Total alkalinity and dissolved inorganic carbon (DIC) were determined using the same methods described in the *Seawater Carbonate Chemistry* section.

Over the course of the collection period three duplicate seawater samples were collected for quality control and to ensure that the collection and analysis methods were accurate. Differences between duplicate measurements were small (on average 4.2 ± 2.0 μmol kg^-1^ for TA and 0.8 ± 0.3 μmol kg^-1^ for DIC), which resulted in small differences in calculated values for pH, *p*CO_2_, Ω_C_ and Ω_A_ (SI Table 1).

*pH control and experimental set up*

Biofilms were developed from April to October 2015 in a flow-through seawater system housed in a 13°C control temperature room at the Portobello Marine Laboratory (PML) at the University of Otago. A total of three independent systems were used, one for each target pH/*p*CO_2_ treatment, namely ambient pH_NIST_ 8.1 (*p*CO_2_ = 384 μatm), pH_NIST_ 7.8 (near future, 2100, *p*CO_2_ = 1108 μatm) and pH_NIST_ 7.4 (extreme, 2300, *p*CO_2_ = 2465 μatm). Each system consisted of a 70L header tank that fed four smaller 10L replicate aquaria (30 cm x 20 cm x 12 cm) at a continuous rate of 1 L min^-1^ (SI Figure 8). Seawater pH was regulated by the direct and controlled bubbling of 100% CO_2_ gas into each header tank using an automatic CO_2_ injection system. Injection rate was controlled through feedback to solenoids by pH_NIST_ calibrated glass electrodes connected to controllers to maintain stabilized pH levels (TUNZE pH/CO_2_ controllers 7074/2, TUNZE AQUA RIENTECHNIK GMBH, Penzberg, Germany).

Macro-filtered seawater was supplied at ambient temperature and pH from the Otago Harbour into each header tank via a 130 µm TAGLINE screen filter. A ball float was used to maintain water level and seawater was mixed in each header tank using a submersible pump before reaching replicate treatment aquaria. Light conditions were controlled by suspending two 120 cm and one 150 cm waterproof, under-verandah light units with twin Alto TLD tube fluorescent bulbs in “cool white” approximately 1 meter above all treatment replicates. Lights were controlled on a timer set to a 10:14h light:dark cycle to maintain consistent light conditions over the duration of the experiment. Comparable *in situ* ecosystems experience diel illuminance fluctuations between 0 to 100,000 lm/m^2^. In this experiment illuminance was monitored daily using a Digitech QM1587 light meter (lm/m^2^) to ensure illuminance was within an acceptable range across treatments (1644 to 2332 lm/m^2^) that emulated *in situ* conditions.

*Seawater Carbonate Chemistry*

Over the course of biofilm development pH was monitored on a daily basis by laboratory technicians using pH_NIST_ calibrated glass electrodes and was recorded approximately every 3-4 days over the duration of the experiment (SI Figure 9). In addition, water samples were collected from the flow-through system approximately every three weeks to track the carbonate system, and results were grouped into two periods: period 1 from May–June 2015, and period 2 from August–October 2015 (SI Table 2). For these measurements duplicate seawater samples were collected from each tank in a 250 mL Duran Schott bottle (for dissolved inorganic carbon, DIC) and a 1L PET bottle (for total alkalinity, TA). All samples were fixed with saturated HgCl_2_ for later analysis.

Alkalinity was measured by potentiometric titration in a closed cell following the method of Dickson et al.[1]. The accuracy of the alkalinity method was determined by the analysis of Certified Reference Material provided by Andrew Dickson from Scripps Institution of Oceanography (SIO) and is estimated to be within ± 2 μmol kg^-1^. DIC was measured by coulometric analysis of evolved gas after the acidification of a seawater sample following the method of Dickson et al. [1].  The accuracy of the DIC method was determined by analysis of Certified Reference Material provided by Andrew Dickson from SIO and is estimated to be within ± 1 μmol kg^-1^.

Values were used as input parameters into the program SWCO_2_ [2] along with temperature (°C) and salinity (ppt) to determine the pH_NIST_, partial pressure of CO_2_ (ρCO_2_, μatm) and the saturation state of calcite (Ω_C_) and aragonite (Ω_A_) for each treatment. Seawater properties were determined using the CO_2_ equilibrium constants given by Mehrbach et al. [3] and refit by Dickson and Millero [4].

*Biofilm development*

Biofilms were developed on glass microscope slides (25mm x 75mm) previously sanded with 800-grade Emory paper, cleaned to remove any chemical residue by soaking in a 10% HCl bath for 48 h, rinsed with deionized, distilled water (Milli-Q, Millipore water) and autoclaved prior to deployment in tanks. Glass microscope slides have previously been shown to be suitable settlement substrates for biofilm development [5-7], particularly over long-term development time frames where temporal shifts diminish the effect of substrate type on community structure

[8,9]. Slides were suspended below water’s surface by custom-built foam trays designed to hold five slides spaced 5 cm apart at a 45° angle perpendicular to flow-through. Each replicate tank held two foam holders approximately 10 cm apart. The upper 3.75 cm^2^ of each slide was secured in the foam holder with the lower 15 cm^2^ exposed to seawater flow. Foam holders were cleaned with Virkon S (DuPont) disinfectant and then thoroughly washed and soaked in Milli-Q water to prevent contamination.

*Microbial biofilm sampling protocol, wet weight biomass and DNA extraction*

Biofilms for weight and microbial community analysis where collected from period 1 (P1) and pigments and settlement assays were collected from period 2 (P2). Biofilm material was carefully removed from both sides of the lower 15 cm^2^ section of each slide (total area = 30 cm^2^) using a sterilised metal laboratory spatula (method adapted from [6]). Samples were deposited in pre-weighed and labeled sterile 1.5mL snap-lock micro-centrifuge tubes and immediately weighed for biofilm wet weight biomass (mg). Tubes were placed in a -4°C freezer for storage until transportation to the Microbiology and Immunology Department, University of Otago in an iced cooler and then placed again into a -20°C freezer until further processing.

Total DNA was extracted from each individual biofilm sample using a MoBio PowerSoil^TM^ DNA Isolation Kit (MO BIO Laboratories Inc., Solana Beach, CA, USA) following manufacturer’s protocol with the following modification: Bead-beating (2 x 15s) cycles was performed using a 2010 GenoGrinder (SPEX SamplePrep, Metuchen, NJ, USA). After extraction, a Nanodrop Spectrophotometer (Thermo Fisher Scientific, Waltham, MA, USA) was used to assess DNA quantity and quality.

*Chlorophyll-a and carotenoid pigment extractions*

The analysis of total Chlorophyll-*a* and carotenoid pigment content was adapted from methods for standard [10] and aquatic biofilm [11] analyses. Glass fiber filters (0.7 μm GF/F, Sterlitech) were used to carefully wipe biofilm material from the lower 15 cm^2^ section of each slide (total area = 30 cm^2^). Chlorophyll-*a* and organic pigments were extracted from filters in 10 ml of 90% acetone over a 48 h period in complete darkness at 4°C. Filters were crushed and agitated with a sterile laboratory spatula upon introduction to acetone. Samples were centrifuged at 10,000g for 10 minutes and chlorophyll-*a* and carotenoid pigment concentration was determined spectrophotometrically (Shimadzu PharmaSpec UV-1700) following procedures described by Parsons et al. [10]. Results are given as mg Chl-*a* per cm^2^ and mg carotenoids per cm^2^ as calculated from the individual biofilm slide surface areas of 30 cm^2^ [11].

*16S rRNA gene sequencing and statistical analysis*

The 16S rRNA gene amplicon sequencing was performed using primers 515F/806R (V4 region of the 16S gene) and the Earth Microbiome Project conditions (Version 4_13)[12]. All samples were sequenced on a single Illumina MiSeq run. Sequences were first processed in Qiime (version 1.9.1) using default parameters[13] including minimum read length of 75 bp, min number of consecutive high quality base calls to include a read as a fraction of the input read length of 0.75, Phred quality score of 3, no ambiguous bases allowed, and no mismatches allowed in primer sequence

[14]. All sequences kept for analysis were 151 bp. Sequence clustering (97% sequence similarity) into Operational Taxonomic Units (OTUs) was done using the SILVA (version 119) reference library[15] and UCLUST[16] following the open-reference Operational Taxonomic Unit (OTU) picking protocol. Taxonomy assignments were done using BLAST against the SILVA database (max-e value = 0.001)[17]. Subsampling and rarefactions (10 times) were performed to equal read depths of 8,000 per sample, and samples below that threshold were removed. After rarefaction, all 10 OTU tables were merged and exported for further processing in R[18]. The rarified biom file was processed using the phyloseq package

[19]. To account for the multiple rarifications (10 total) abundances a mean was calculated (dividing by 10) and results were rounded to whole integers using the *transform_sample_counts()* command. Taxa (OTUs) with less than 1 count were deleted using the *prune_taxa()* command. Alpha diversity (Shannon and richness) were calculated using the *estimate_richness()* command.

The NMDS plot was created using a Bray-Curtis distance matrix through “phyloseq” and “vegan”[20] packages. Significant treatment and age effects where determined using an Anosim test. To determine samples forming statistically significant groups, a cluster analysis was performed using the pvclust package (method = Ward; distance matrix = Bray-Curtis; bootstrap value, n = 1000)[21]. Significant groups (representing 95% confidence) were marked with boxes (red). All data analyzed in this paper along with analysis code can be found at: https://github.com/semorales/Nelson_OA_2017.

*Galeolaria hystrix collection, spawning and settlement experiments*

Ripe *Galeolaria hystrix* were collected during September 2015 at low tide from a rocky shore adjacent to the Portobello Marine Laboratory, located at 45° 49’ 42.92” S, 170° 38’ 28.89” E. Adults were stripped spawned, fertilised and larvae reared to settlement using methods described by Nelson et al. [22] After 16 to 19 days of development, larvae were morphologically competent to begin settlement. Competent larvae are demersal and sink to the bottom of the jar. Culture jars were left for 2h, after which 40 demersal larvae were sampled from the bottom and examined under a compound microscope to ensure larvae were competent for settlement. All samples showed 100% larval competency prior to the settlement assays. This was determined by the appearance of a transverse or head constriction [22].

Settlement assays were conducted on biofilms developed on glass slides for 23-days and 60-days in the flow-through system during period 2 (P2) as previously described. All assays compared biofilms reared in three-pH_NIST_ treatments (ambient pH, pH 7.8 and pH 7.4). Each dish was filled with 70 ml of 13°C ambient, filter seawater (FSW 0.1μm) that was sampled for carbonate chemistry analysis using total alkalinity (TA) and dissolved inorganic carbon (DIC) measurements. Four replicate biofilms from each pH treatment were place horizontally along the bottom of plastic petri dishes (88mm diameter, 12 mm height). Thirty larvae were carefully pipetted from the bottom of the culture jar into each petri dish and covered with a plastic top to prevent evaporation. Replicates were left in a randomized order on a white tray in a 15°C CT room with artificial lights set to a 10h:14h light:dark cycle. Larvae were not fed over the duration of the settlement trials. Settlement was measured and scored at two time periods; 24h and 48h after the introduction of larvae.

Ice was placed on the white tray to maintain temperature while petri dishes were inspected under a dissecting microscope. No more than 5 minutes was spent on each individual biofilm to prevent experimental bias during observations and extreme temperature fluctuations caused by over exposure to the microscope light source. Larvae that had reached 1) attachment, 2) metamorphosis and/or 3) juvenile growth were scored and categorised as having achieved settlement success. Settlement data was converted into proportion data (% settlement success).

*Statistical analysis for settlement assays*

Settlement data was converted into proportion data (% settlement). All percentage data was transformed using an arcsine square-root transformation. Data was assessed for outliers and normality using the visual inspection of boxplots for values greater than 1.5 box-lengths, Q-Q plots and calculated studentised residuals. Overall, three outliers were detected and replaced with the next highest/lowest observation from that treatment. The Shapiro – Wilk’s Test of Normality (p>0.05), Levene’s Test of Equality of Error Variances (p>0.05) and Box’s Test of Equality of Covariance Matrices (p>0.05) were used to confirm normality, homogeneity of variance and homogeneity of covariance when needed. Homogeneity of variances was present in all data subsets except for one. In this case heterogeneous variances were compared using the Welch ANOVA testing for equal means, allowing for unequal variances. Since little to no settlement was recorded on the negative controls (sterile glass slides), they were removed from ANOVA analyses due to violations of normality and equality of variances. However, when settlement was present it is included in the graphic representations of the data.

We used an ANOVA and Tukeys HSD test to test for significant effects. To correlate abundance of different organisms to pH we used a Spearmans correlation. All analysis where performed in R with code available (see above).

*Limitations*

There were a number of experimental limitations that the authors could not address due to budget, equipment and laboratory restrictions. Given the well-established physical and chemical sensitivity of biofilms, direct chemical manipulation of seawater pH was not appropriate. However, the authors did not possess the equipment or budget required to create completely independent, randomized treatments with direct injection of CO_2_ gas into each replicate, as outlined as “best practice” by Cornwall & Hurd [23]. Instead a single header tank was used for each treatment (termed “B-4 randomized but with interdependent treatment replicates”) and statistical practices were used to account for pseudo-replication in biofilm analysis.

Additionally, due to size and scope of this complex MSc experiment, equipment availability and technician support required that biofilms developed for microbial analysis (P1) and chlorophyll-a, carotenoid and invertebrate settlement analysis (P2) be grown over separate timeframes. It is well established that seasonality affects microbial community composition, however community scale shifts driven by ocean acidification are detectable regardless of season [24]. Given this limitation, it is important to note that results from the microbial analysis (P1) can not be directly compared as “cause and effect” to results from chlorophyll-a, carotenoid and invertebrate settlement analysis (P2).

Finally, while shallow containers, such as petri dishes, pose concerns with regards to the proximity of the larvae to the settlement surface and detection of biochemical cues; in the context of this experiment authors selected the most appropriate containers that matched *in situ* environmental conditions and practical restrictions. The pilot species, *G. hystrix,* are suspension feeders and typically settle as individuals in the shallow water of low intertidal zones near the sampling site [22]. *In situ* settlement conditions (such as long exposures to shallow water) were used to inform container selection, as well as the need to meet the practical (able to fit under a dissecting microscope) and budgetary requirements of the experimental design.

**References**

1. Dickson, A. G., Sabine, C. L. & Christian, J. R. 2007 Guide to best practices for ocean CO2 measurements., 191.

2. Hunter, K. A. 2007 SWCO2 Seawater CO2 equilibrium calculations. *http://neon.otago.ac.nz/research/kah/software/swco2/index.html*.

3. Mehrbach, C., Culberson, C. H., Hawley, J. E. & Pytkowicx, R. M. 2003 Measurement of the Apparent Dissociation Constants of Carbonic Acid in Seawater at Atmospheric Pressure. *Limnol. Oceangr.* **18**, 897–907. (doi:10.4319/lo.1973.18.6.0897)

4. Dickson, A. G. & Millero, F. J. 1987 A comparison of the equilibrium constants for the dissociation of carbonic acid in seawater media. *Deep Sea Research Part A. Oceanographic Research Papers* **34**, 1733–1743.

5. Witt, V., Wild, C., Anthony, K. R. N., Diaz-Pulido, G. & Uthicke, S. 2011 Effects of ocean acidification on microbial community composition of, and oxygen fluxes through, biofilms from the Great Barrier Reef. *Environ Microbiol* **13**, 2976–2989. (doi:10.2216/08-99.1)

6. Witt, V., Wild, C. & Uthicke, S. 2011 Effect of substrate type on bacterial community composition in biofilms from the Great Barrier Reef. *FEMS Microbiology Letters* **323**, 188–195. (doi:10.1111/j.1574-6968.2011.02374.x)

7. Witt, V., Wild, C. & Uthicke, S. 2012 Interactive climate change and runoff effects alter O2 fluxes and bacterial community composition of coastal biofilms from the Great Barrier Reef. *Aquat. Microb. Ecol.* **66**, 117–131. (doi:10.3354/ame01562)

8. Huggett, M. J., Nedved, B. T. & Hadfield, M. G. 2009 Effects of initial surface wettability on biofilm formation and subsequent settlement of Hydroides elegans. *Biofouling* **25**, 387–399. (doi:10.1080/08927010902823238)

9. Chung, H. C., Lee, O. O., Huang, Y.-L., Mok, S. Y., Kolter, R. & Qian, P.-Y. 2010 Bacterial community succession and chemical profiles of subtidal biofilms in relation to larval settlement of the polychaete Hydroides elegans. *The ISME Journal* **4**, 817–828. (doi:10.1038/ismej.2009.157)

10. Parsons, T. R., Maita, Y. & Lalli, C. M. 1984 Determination of chlorophylls and total carotenoids: spectrophotometric method. *Parsons, TR, Y. Maita and CM Lalli. A manual of chemical and biological methods for seawater analysis. Pergamon Press, Oxford*, 101–112.

11. Artigas, J., Roman, A. M. & Sabater, S. 2015 Nutrient and enzymatic adaptations of stream biofilms to changes in nitrogen and phosphorus supply. *Aquat. Microb. Ecol.* **75**, 91–102. (doi:10.3354/ame01745)

12. Caporaso, J. G. et al. 2012 Ultra-high-throughput microbial community analysis on the Illumina HiSeq and MiSeq platforms. *The ISME Journal* **6**, 1621–1624. (doi:10.1038/ismej.2012.8)

13. Caporaso, J. G. et al. 2010 QIIME allows analysis of high- throughput community sequencing data. *Nature Methods* **7**, 335–336. (doi:10.1038/nmeth0510-335)

14. Bokulich, N. A., Subramanian, S., Faith, J. J., Gevers, D., Gordon, J. I., Knight, R., Mills, D. A. & Caporaso, J. G. 2013 Quality-filtering vastly improves diversity estimates from Illumina amplicon sequencing. *Nature Methods* **10**, 57–59. (doi:10.1038/nmeth.2276)

15. Quast, C., Pruesse, E., Yilmaz, P., Gerken, J., Schweer, T., Yarza, P., Peplies, J. & Glockner, F. O. 2012 The SILVA ribosomal RNA gene database project: improved data processing and web-based tools. *Nucleic Acids Research* **41**, D590–D596. (doi:10.1093/nar/gks1219)

16. Edgar, R. C. 2010 Search and clustering orders of magnitude faster than BLAST. *Bioinformatics* **26**, 2460–2461. (doi:10.1093/bioinformatics/btq461)

17. Altschul, S. F., Gish, W., Miller, W., Myers, E. W. & Lipman, D. J. 1990 Basic local alignment search tool. *Journal of Molecular Biology* **215**, 403–410. (doi:10.1016/S0022-2836(05)80360-2)

18. R Development Core Team 2008 R: A language and environment for statistical computing. *R Foundation for Statistical Computing, Vienna, Austria*

19. McMurdie, P. J. & Holmes, S. 2013 phyloseq: an R package for reproducible interactive analysis and graphics of microbiome census data. *PLoS ONE* **8**, e61217. (doi:10.1371/journal.pone.0061217)

20. Oksanen, J. et al. 2013 vegan: Community Ecology Package. *Community ecology …*. (doi:10.2307/41739370?ref=search-gateway:f5808b76cab0ff484aa451797660a8f5)

21. Suzuki, R. & Shimodaira, H. 2006 Pvclust: an R package for assessing the uncertainty in hierarchical clustering. *Bioinformatics* **22**, 1540–1542. (doi:10.1093/bioinformatics/btl117)

22. Nelson, K. S., Liddy, M. & Lamare, M. D. 2017 Embryology, larval development, settlement and metamorphosis in the New Zealand Serpulid Polychaete Galeolaria hystrix. *Invertebrate Reproduction & Development* **2**, 1–11. (doi:10.1080/07924259.2017.1318183)

23. Cornwall, C. E., & Hurd, C. L. (2015). Experimental design in ocean acidification research: problems and solutions. *ICES Journal of Marine Science*, *73*(3), 572-581.

24. Krause, E., Wichels, A., Giménez, L., Lunau, M., Schilhabel, M. B., & Gerdts, G. (2012). Small changes in pH have direct effects on marine bacterial community composition: a microcosm approach. *PloS one*, *7*(10), e47035.
